# Monsters in the uterus: teratoma-like tumors in senescent *C. elegans* result from a parthenogenetic quasi-program

**DOI:** 10.18632/aging.101486

**Published:** 2018-06-19

**Authors:** Hongyuan Wang, Zhizhou Zhang, David Gems

**Affiliations:** 1 School of Chemistry and Chemical Engineering, Harbin Institute of Technology, Harbin 150001, China; 2 Institute of Healthy Ageing, University College London, London WC1E 6BT, UK

**Keywords:** aging, *C. elegans*, quasi-programs, teratoma, tumor

Genes such as the *daf-2* insulin/IGF-1 receptor and the *daf-16* FoxO transcription factor dramatically affect *C. elegans* lifespan, but understanding how they do so has proved a difficult nut to crack. Solving this is important, since the biology involved is that of aging itself, which is the cause of the diseases that most of us die of. Many prior investigations have been gene focused, identifying genes that affect lifespan, and studying the molecular biology of the proteins that they encode. An alternative approach is to focus on pathology, on the assumption that senescent *C. elegans*, like old people, die as the result of specific diseases of aging. From this it follows that genes affecting lifespan act by altering such pathologies, which represent a missing link in the causal chain between gene product and lifespan. In terms of understanding aging, a key question is: what are the mechanisms that generate senescent pathologies? Arguably, the identity of the pathologies that actually limit life in worms is of secondary importance.

To illustrate: consider two major sites of senescent pathology in *C. elegans*: the pharynx and the uterus. Mechanical senescence of the pharynx (the muscular pump in the head that sucks in and grinds up the worm's microbial diet) leads to lethal infection that kills ~40% of wild type worms [1]. By contrast, the large tumors that grow in the *C. elegans* uterus, formed from clusters of unfertilized oocytes, have no effect on lifespan [2]. Yet uterine tumor formation is clearly part of *C. elegans* senescence, and cancer contributes to mortality in old people; moreover, both uterine tumors and colon cancer are treatable with the drug 5-fluorodeoxyuridine (floxuridine).

Several facts about the origins of uterine tumors have long been known. In *C. elegans* hermaphrodites, oocytes are fertilized using a limit stock of self sperm. After sperm depletion, unfertilized oocytes enter the uterus inappropriately, and become polyploid and hypertrophic, such that the swollen uterus grows to fill much of the worm mid-body [3]. This abnormal growth (seen in all animals) appears to reflect futile attempts by the unfertilized oocytes to develop into embryos, and is promoted by DAF-2 [4].

This simple pathobiology provides some useful lessons, for example about theories of aging [5]. Here the wild-type *daf-2* insulin/IGF-1 receptor gene is exhibiting antagonistic pleiotropy (AP), promoting development first of germline and then of tumors. Evolutionary biologists like G.C. Williams, originator of the AP theory, envisaged AP acting on simple, structural genes with narrow phenotypic effects [6]. Consequently, they were at first surprised by the discovery of genes like *daf-2* apparently affecting the entire aging process. This paradox was partially resolved when it was demonstrated that *daf-2* and *daf-16* influence large numbers of other genes. These included many genes involved in somatic maintenance, consistent with the notion that a major cause of aging is molecular damage accumulation, and the disposable soma interpretation of AP action. However, a better fit with uterine tumor pathobiology is M.V. Blagosklonny's concept of the quasi-program: a complex, developmental program that runs on, resulting in the development of pathology [7]. By analogy, an oven programmed to roast a leg of lamb that remains on for too long leads to it becoming over-cooked. Here pathology results not passively from damage accumulation or failure of homeostasis, but rather actively from gene action: not from loss of function, but hyper-function [7].

One reason sometimes given for not studying native senescent pathology in *C. elegans* is their assumed lack of relevance to mammalian disease biology. In fact, worm uterine tumors are etiologically similar to ovarian teratomas, which occur in mammals of reproductive age, including mice and women. Teratomas are an unpleasant looking, yet benign form of congenital tumor (hence teratoma, Greek: monster growth) that form as the result of the expression of programs of embryonic development within somatic tissues. Ovarian teratomas form sacs containing multiple tissues (skin, cartilage, hair). Both ovarian teratomas and worm uterine tumors result from embryogenetic quasi-programs in abnormal diploid cells resulting from a failure of meiosis II [5]. Notably, older *C. elegans* uterine tumors show expression of markers of later embryogenesis, though no actual differentiated cells, hence are teratoma-like [5]. The presence here of broadly similar mechanism demonstrates the potential biomedical utility of studying wild-type pathophysiology in *C. elegans*. Also, it is difficult to look at aging in the same way once one has realized that it is a broadly similar sort of pathology to teratoma (insofar as senescence is caused by quasi-programs).

## References

[1] Zhao Y, et al. Nat Commun. 2017; 8:15458. 10.1038/ncomms15458 28534519 PMC5457527

[2] Riesen M, et al. Aging (Albany NY). 2014; 6:98–117. 10.18632/aging.100638 24531613 PMC3969279

[3] McGee MD, et al. Aging (Albany NY). 2012; 4:256–69. 10.18632/aging.100448 22562940 PMC3378273

[4] Ezcurra M, et al. Curr Biol. 2018 Epub ahead of print.

[5] Wang H, et al. Aging and Mechanisms of Disease. 2018; 4:6. 29928508 10.1038/s41514-018-0025-3 PMC5998035

[6] Williams GC. Evolution. 1957; 11:398–411.

[7] Blagosklonny MV. Cell Cycle. 2006; 5:2087–102. 10.4161/cc.5.18.3288 17012837

